# Modulatory Effects of Alpha- and Gamma-Tocopherol on the Mitochondrial Respiratory Capacity and Membrane Potential in an *In Vitro* Model of Alzheimer’s Disease

**DOI:** 10.3389/fphar.2021.698833

**Published:** 2021-11-22

**Authors:** Aslina Pahrudin Arrozi, Wan Zurinah Wan Ngah, Hanafi Ahmad Damanhuri, Suzana Makpol

**Affiliations:** Department of Biochemistry, Faculty of Medicine, Universiti Kebangsaan Malaysia Medical Centre, Kuala Lumpur, Malaysia

**Keywords:** tocopherol, amyloid precursor proteins, respiratory capacity, membrane potential, Alzheimer’s disease

## Abstract

Increased amyloid-beta (Aβ) and amyloid precursor protein (APP) in the brains of Alzheimer’s disease (AD) patients are common pathological hallmarks mediating the disease progression. Growing evidence also suggests that mitochondrial abnormalities are an early feature in the pathogenesis of AD. Intervention with antioxidants has received great interest as a molecular strategy for the manipulation of mitochondrial function. Our previous preliminary study using *in vitro* cell models expressing different types of APP demonstrated that treatment with alpha-tocopherol (ATF) or gamma-tocopherol (GTF) modulates mitochondrial function by reducing mitochondrial reactive oxygen species (ROS), increasing the production of ATP and preventing apoptosis events, especially in cells expressing the mutant APP form. Thus, we hypothesized that ATF or GTF treatment might also alter mitochondrial metabolic pathways such as oxidative phosphorylation. The present study aimed to investigate the role of ATF and GTF in modulating mitochondrial oxidative metabolism using high-resolution respirometry. Our results showed that both ATF and GTF increased the respiratory capacity and membrane potential in the ROUTINE and OXPHOS_CI-LINKED_ states as well as complex IV enzyme activity in wild-type and mutant APP-overexpressing SH-SY5Y cells. Although preliminary, these findings indicate that ATF and GTF modulate mitochondrial oxidative metabolism in APP-overexpressing cells and, in part, may contribute to the planning of strategies for utilizing vitamin E isomers against mitochondrial-related diseases such as AD.

## Introduction

The accumulation of insoluble amyloid-beta (Aβ) protein in extracellular amyloid plaques in the brains of Alzheimer’s disease (AD) patients is well known as the classical pathological hallmark of the disease (Tiwari et al., 2019) together with the presence of neurofibrillary tangles (NFTs) (Gao et al., 2018). In addition to abnormalities in protein aggregation, other pathological changes are seen in this condition, such as alterations in mitochondrial function (Bell et al., 2021). A recent study showed that overexpression of the β-secretase-derived APP-CTF fragment (C99) in neuroblastoma SH-SY5Y cells triggers excessive mitochondrial morphology alterations associated with enhanced mitochondrial reactive oxygen species production independent of Aβ (Vaillant-Beuchot et al., 2021). Another study showed that overexpression of the human amyloid precursor protein (APP) with the familial Swedish mutation (APPswe) in a mouse neuroblastoma cell line (N2A) altered the structure and function of mitochondria-associated membranes, mitochondrial dynamics, biogenesis and protein import as well as the stress response (Fernandes et al., 2021). Previously, our group showed that the level of reactive oxygen species (ROS) in the mitochondria, activity of complex V enzyme, and cyclophilin D (CypD) and pro-caspase 3 protein expression as well as cytochrome c release were increased, followed by a decrease in ATP levels in SH-SY5Y cells overexpressing the mutant APP form (Pahrudin Arrozi et al., 2020), suggesting that the mitochondrial function in these *in vitro* models of AD was also altered. Thus, we hypothesized that mitochondrial metabolic pathways, such as oxidative phosphorylation and membrane potential, were also altered, as mitochondrial oxidative metabolism is the major site of ATP synthesis.

Several previous studies have demonstrated the tremendous role of alpha-tocopherol (ATF) as a radical-scavenging antioxidant to protect cells against ROS (Ulatowski and Manor, 2015) and as a signalling molecule that influences the expression of genes involved in disease development (Pédeboscq et al., 2012; Zingg, 2019). ATF is the most widely studied vitamin E isomer, as it has the highest biological activity in the body (Reboul, 2017) and is related to vitamin E deficiency syndrome (Jilani and Iqbal, 2018). In addition to ATF, other isomers of vitamin E, such as gamma-tocopherol (GTF), have also demonstrated unique functions in preventing oxidative stress in the brain related to reactive nitrogen species (RNS) (Williamson et al., 2002) regardless of their low bioavailability in the body (Ashley et al., 2019). Furthermore, a previous study also demonstrated that lower brain GTF in combination with higher ATF was associated with higher AD neuropathology (Morris et al., 2015), and higher GTF levels were found to be associated with higher presynaptic protein levels in the elderly human midfrontal cortex (de Leeuw et al., 2020), supporting the important role of GTF in the brain.

Studies on the role of ATF or GTF treatment on mitochondria are still limited. A recent *in vitro* study showed that treatment with different vitamin E isoforms, including ATF, had protective effects by reducing oxidative stress and increasing metabolic activity *via* oxidative metabolism in a human endothelial cell line under conditions mimicking sepsis (Minter et al., 2020). In addition, an *in vivo* study with preadministration of high-dose ATF showed significantly improved memory and mitochondrial membrane potential impairment as well as reduced ROS levels in the rat hippocampus induced with lactacystin, a proteasome inhibitor (Nesari et al., 2019). Our previous preliminary study revealed that treatment with ATF or GTF for 24 h reduced the Aβ42 and mitochondrial ROS levels, increased the activity of complex V enzyme and ATP levels, and prevented apoptotic events in SH-SY5Y cells overexpressing mutant APP (Pahrudin Arrozi et al., 2020), suggesting the protective effects of vitamin E on mitochondria. Therefore, in the present study, we aimed to investigate the potential effects of ATF and GTF in modulating mitochondrial oxidative metabolism to further elucidate the protective role of both vitamin E isomers, which may contribute to strategies against mitochondrial-related diseases such as AD.

## Materials and Methods

### Development of SH-SY5Y Cells Overexpressing Wild-type and Mutant APP Genes

The development of SH-SY5Y cells stably expressing the wild-type or mutant APP gene was described in our previous study (Pahrudin Arrozi et al., 2017). Briefly, SH-SY5Y cells were transfected with three different plasmids carrying the wild-type (WT), Swedish (Swe), or Swedish/Indiana (Swe/Ind) form of the APP gene using Lipofectamine 3000 (Invitrogen, Carlsbad, United States). After 72 h of transfection, the positive green fluorescent protein (GFP)-expressing cells were selected using selection medium containing 400 μg/ml geneticin (G418) (Gibco, Calrsbad, United States). The steady state APP gene and protein expression as well as the production of secreted Aβ42/Aβ40 were measured for verification. The methodology and results of these evaluations have been published, whereby the levels of APP mRNA and protein expression were increased in all cells stably expressing the different types of APP genes, while the ratio of Aβ42/40 was found to be increased in the following order: SH-SY5Y-APP WT < SH-SY5Y-APP Swe < SH-SY5Y-APP Swe/Ind (Pahrudin Arrozi et al., 2017).

### Cell Culture

The nontransfected SH-SY5Y cells were grown in complete culture medium (CCM) containing a 1:1 ratio of Dulbecco’s modified Eagle’s medium (DMEM) and Ham’s F-12 medium with 1% penicillin/streptomycin (Gibco) and 10% foetal bovine serum (FBS) (HyClone, Utah, United States). The stably transfected SH-SY5Y cell lines were grown in CCM without penicillin/streptomycin but with 400 μg/ml geneticin (Gibco). All types of cells were cultured in a humidified atmosphere of 5% CO_2_ at 37°C.

### Treatment of Cells With Tocopherol Isomers

Stock solutions of ATF (98.9% pure d-ATF) and GTF (95% pure d-GTF) (ChromaDex, California, United States) were freshly prepared in 100% ethanol (EtOH) to a final stock solution concentration of 0.5 M and stored at −20°C for not more than 1 month. Prior to treatment, 15 µl of each isomer from the stock solution was incubated overnight with 20 µl of FBS at 37°C and then diluted to 0.1 M with 18 µl of CCM and 21 µl of 100% ethanol. The isomer solution was further diluted to 0.05 M with a 72 µl mixture of CCM and 100% ethanol in a 1:1 ratio (50% ethanol). Subsequently, the isomer solution was diluted to 0.025 M with 146 µl of CCM only. Next, 60 µl from the 0.025 M isomer stock solution was removed and transferred into a new microcentrifuge tube. Then, 240 µl of CCM was added to prepare the 0.005 M stock solution. Two more stocks solutions (0.004 and 0.00025 M) were prepared by taking out 80 and 5 µL from the 0.005 M stock solution and combining them with 20 and 95 µL of CCM, respectively. Finally, the desired working concentrations of each isomer (5, 80, and 100 µM) were prepared from their respective stock solution (0.00025, 0.004, and 0.005 M), to give the final concentration of each tocopherol isomer in the culture media added to the cells. The final concentrations of ethanol and FBS were kept constant at 0.1 and 0.027%, respectively (calculated from the overnight incubation with 20 µL of FBS). Tocopherol treatment was carried out for 24 h. The cytotoxicity assay was performed in our previous study, and the results showed that ATF or GTF at concentrations from 1 to 100 µM were not toxic to the cells (Pahrudin Arrozi et al., 2020).

### Chemicals and Reagents Used in the Oxygraph-2k Experiment

All chemicals and reagents used in Oxygraph-2k (O2k), such as ethylene-glycol-bis(β-aminoetyl ether)-N,N,N′,N′-tetraacetic acid (EGTA), magnesium chloride hexahydrate (MgCl_2_6H_2_O), lactobionic acid, taurine, potassium dihydrogen phosphate (KH_2_PO_4_), HEPES, D-sucrose, free fatty acid bovine serum albumin (BSA), sodium pyruvate (P), malate (M), glutamate (G), succinate (S), cytochrome c, catalase, adenosine 5′-diphosphate (ADP), carbonyl cyanide 3-chlorophenylhydrazone (CCCP), rotenone, antimysin-A, digitonin, safranin, ascorbate, tetramethyl-p-phenylenediamine dihydrochloride (TMPD) and sodium azide (NaN_3_), were from Sigma–Aldrich, Missouri, United States. The preparation of all the substances is described in Supplementary Table S1.

### Simultaneous Determination of the Mitochondrial Respiratory Capacity and Membrane Potential

The simultaneous determination of the respiratory capacity and membrane potential was carried out using an Oxygraph-2k (O2k) (Oroboros Instruments, Innsbruck, Austria) according to the standard protocol as elaborated previously (Krumschnabel et al., 2014; Lemieux et al., 2017). Briefly, at the beginning of each experiment, the O2k chambers were filled with 2.5 ml of Mir05 solution (Supplementary Table S2), excess solution was removed (final volume of Mir05 in the chamber was ∼2.0 ml), calibrated with air at 37°C (9.72 µM O2/kPa) and constantly stirred using white PVDF-coated stirrers at 750 rpm. Each group of cells was trypsinized, and the cell pellet was then resuspended in 300 µL of Mir05 solution. The number of cells was calculated for normalization, and the cell suspension was titrated into chambers. Substrate–uncoupler–inhibitor titration (SUIT) protocols were then applied for the simultaneous determination of mitochondrial respiratory capacity and membrane potential analysis (Supplementary Figure S1).

After cell permeabilization with digitonin, nonphosphorylating LEAK respiration (LEAK_PM_) was induced by adding the CI-linked substrates pyruvate (5 mM) and malate (2 mM). Subsequently, the OXPHOS capacity of CI-linked activity (OXPHOS_CI-LINKED_) was measured after the addition of saturating concentrations of ADP (2.5 mM), cytochrome c (10 µM) and glutamate (10 mM). The OXPHOS capacity with combined CI and CII-linked substrates (OXPHOS_CI-CII-LINKED_) was assessed by the addition of succinate (10 mM). Stepwise titration of CCCP (3 steps; 0.5, 1 and 1.5 µM), 0.5 μM) leads to proton leakage through the inner mitochondrial membrane and was used to measure the capacity of the electron transfer system (ETS), which is the noncoupled state at the optimum uncoupler concentration for maximum oxygen flux. Subsequent inhibition of CI by rotenone (0.5 μM) provided measurement of the CII-linked ETS capacity (ETS_CII-LINKED_). To control for other oxygen-consuming processes, CIII was inhibited by antimycin A (2.5 µM). The resulting residual oxygen consumption (ROX) reflects oxygen consumption from undefined sources and was subtracted from mitochondrial respiratory states. Meanwhile, for membrane potential, the safranin signal was corrected manually using the value of cytochrome c acquired when injected into the chamber alone. The respiratory flux was expressed per million cells (pmol/s/mill cells). The fluorescence of the safranin signal was detected at an excitation wavelength of 495 nm and emission wavelength of 587 nm. The safranin signal (ampere) was inversely proportional to the membrane potential. The membrane potential was calculated as 1/Amp. Each experiment was repeated three times.

### Determination of Complex IV Enzyme Activity

Determination of complex IV enzyme activity was carried out using Oxygraph-2k (O2k) (Oroboros Instruments, Innsbruck, Austria). At the beginning of each experiment, 5 µL of 112,000 μ/mL catalase solution was titrated into O2k chambers filled with 2.5 ml Mir05 (final volume of Mir05 in the chamber was ∼2.0 ml), calibrated with air at 37°C (9.72 µM O_2_/kPa) and constantly stirred using white PVDF-coated stirrers at 750 rpm. Each group of cells was trypsinized, and the cell pellet was then resuspended in 300 µL of Mir05. The number of cells was calculated for normalization followed by titration protocols (Supplementary Figure S2). All substance preparations are presented in Supplementary Table S3. The final concentrations of all the substances were as follows: digitonin (0.01 mg/ml), rotenone (0.5 µM), CCCP (0.5 µM), ascorbate (2 mM), TMPD (0.5 mM), cytochrome c (10 µM), and NaN_3_ (0.2 M). The value of oxygen flux was corrected automatically by Datlab 7 software using the value recorded after the inhibition of complex IV activity due to substrate autooxidation. Each experiment was repeated three times.

### Statistical Analysis

Statistical analyses were performed using GraphPad PRISM v.8 software (GraphPad Software, La Jolla, California, United States). Data obtained were expressed as the mean ± SD. Statistical comparisons between groups were performed using one-way ANOVA followed by the Sidak post hoc test. The statistical significance of all tests was set at *p* < 0.05.

## Results

### An Abnormally Higher Respiratory Capacity and Membrane Potential Was Found in SH-SY5Y Cells Overexpressing APP Wt or APP Swe but Was Impaired in APP Swe/Ind Relative to the Non-transfected SH-SY5Y Control

**FIGURE 1:**
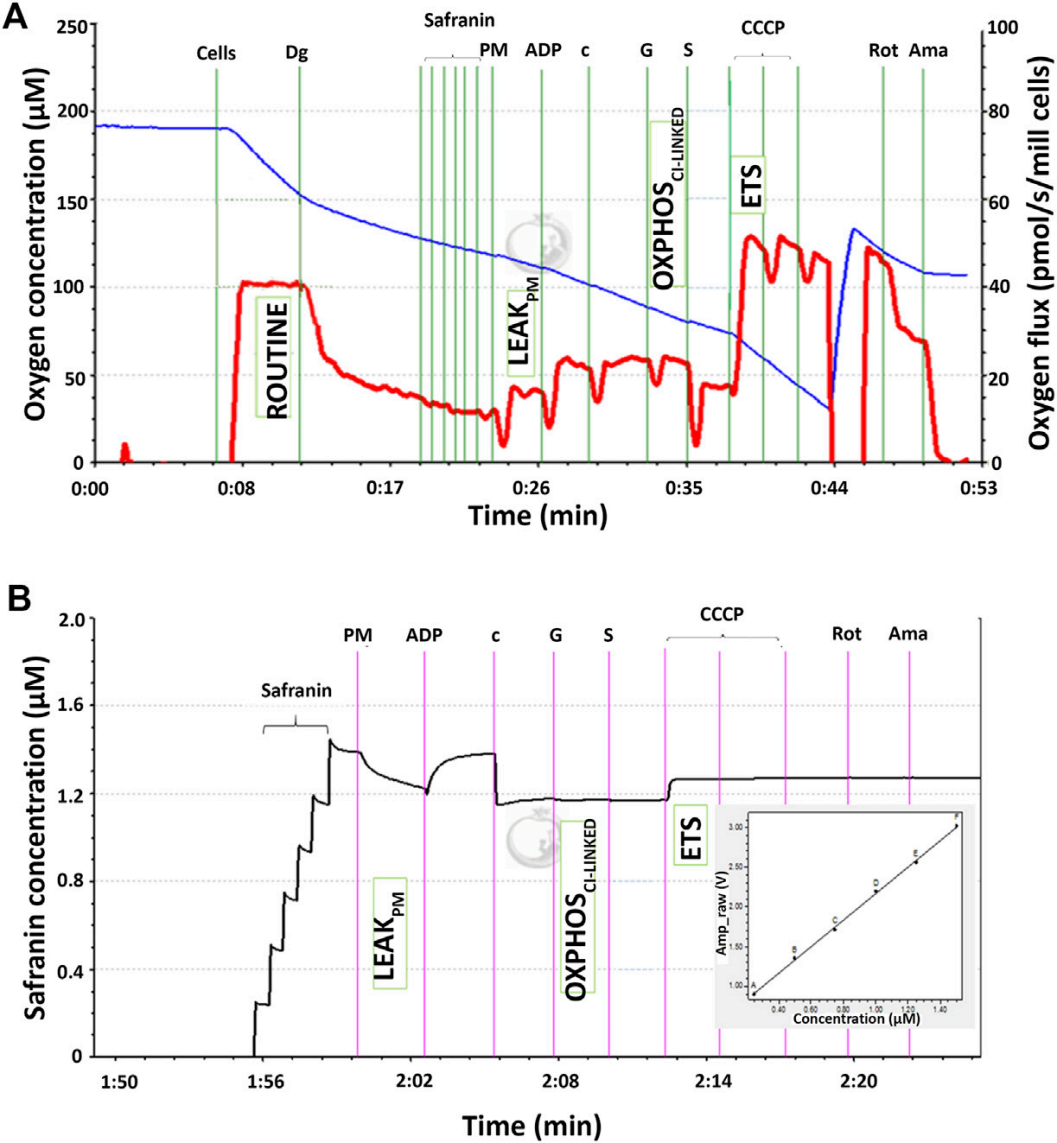
Qualitative data for simultaneous high-resolution respirometry analysis of **(A)** the respiratory capacity at four selected mitochondrial bioenergetics states: ROUTINE, LEAK_PM_, OXPHOS_CI-LINKED_, and ETS expressed as the oxygen flux (pmol/s/mill cells) represented by the red line and **(B)** membrane potential at three selected mitochondrial bioenergetic states: LEAK_PM_, OXPHOS_CI-LINKED_, and ETS. The black line corresponds to the safranin concentration in the chamber medium and is directly proportional to the safranin signal expressed as amperes (amp). The membrane potential was inversely proportional to the safranin signal and thus calculated as 1/amp.

The mitochondrial respiratory capacity and membrane potential were measured simultaneously in this study. Figure 1A shows qualitative data of high-resolution respiration (HRR) using an O2k respirometer with the SUIT protocol. The red line represents the oxygen flux to indicate the volume of oxygen converted by the cells per time per number of cells (pmol/s/mill cells), whereas the blue line represents the level of oxygen concentration inside the chamber. Figure 1B shows the signal of safranin in the chamber medium represented by the black line. The concentration of safranin is directly proportional to the safranin signal (amp) but inversely proportional to the membrane potential.

There were four respiratory capacities measured in the present study including ROUTINE, respiratory capacity in living cells controlled by cellular energy demand, energy turnover and the degree of coupling to phosphorylation, whereas the other three respiratory capacities were measured in cells that selectively permeabilized the plasma membrane of cells while the mitochondrial membrane remain intact thus allowing direct access to the mitochondria, which then required the supply from exogenous substrates/substances to measure mitochondrial respiration. The mitochondrial respiratory capacities are LEAK_PM_, the respiratory capacity by which cation cycling and electron leakage lead to heat production instead of biochemical work. This state was measured in the presence of reducing substrates such as pyruvate and malate but absence of ADP, a component described as the non-phosphorylating resting state when ATP synthase is not active; OXPHOS_CI-LINKED_, the respiratory capacity supported by NADH-generating substrates (pyruvate, malate and glutamate) through complex I to the Q-junction, with further electron transfer through complex III and complex IV to oxygen coupled with phosphorylation in the presence of saturating ADP, a component described as oxidative phosphorylation; and ETS, the respiratory capacity of mitochondria measured as oxygen consumption in the noncoupled state supported by NADH-generating substrates and succinate at optimum uncoupler (CCCP) concentration and is not limited by the capacity of the phosphorylation system, a component described as the electron-transfer-pathway capacity.

**FIGURE 2:**
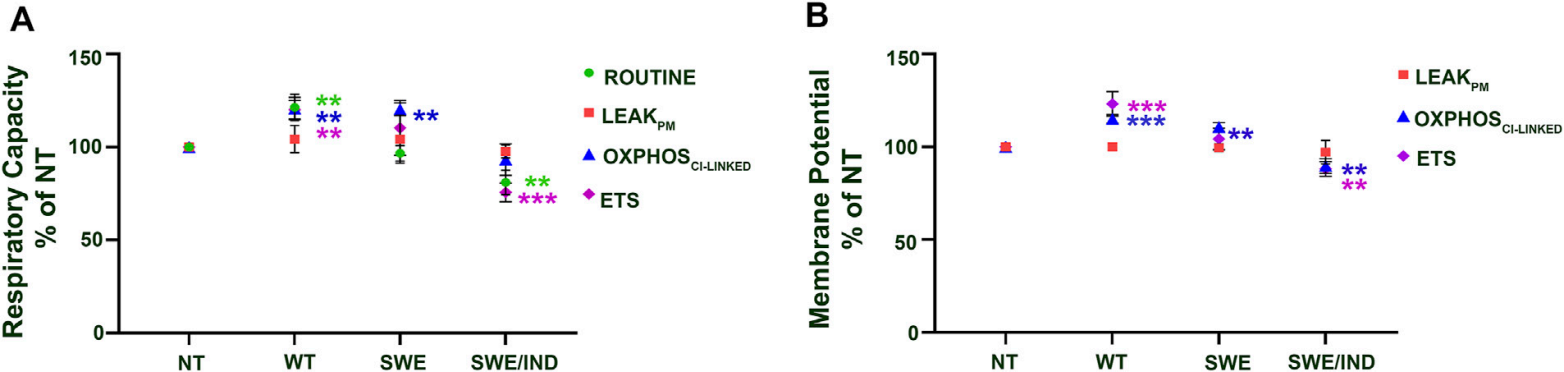
Comparison between SH-SY5Y cells overexpressing different types of APP (WT, Swe and Swe/Ind) at different mitochondrial bioenergetic states (ROUTINE, LEAK_PM_, OXPHOS_CI-LINKED_ and ETS) expressed as % of nontransfected control (NT). **(A)** Respiratory capacity and **(B)** membrane potential. **: *p* < 0.01, ***: *p* < 0.001. Data are expressed as the mean ± SD, (N = 3).

At the baseline level without treatment with ATF and GTF, relative to the nontransfected control, the APP WT cells showed significantly higher respiratory capacity in the ROUTINE, OXPHOS_CI-LINKED,_ and ETS states, whereas APP Swe cells showed significantly higher respiratory capacity only in the OXPHOS_CI-LINKED_ state (Figure 2A). Meanwhile, for APP Swe/Ind cells, the respiratory capacity ROUTINE and ETS states were significantly lower relative to the nontransfected control (Figure 2A). In parallel to higher/lower respiratory capacity, the membrane potential was also higher in APP WT but lower in APP Swe/Ind at OXPHOS_CI-LINKED_ and ETS states relative to the non-transfected control (Figure 2B). However, in APP Swe cells, the membrane potential was higher only in the OXPHOS_CI-LINKED_ state relative to the nontransfected control (Figure 2B). There was no significant difference in respiratory capacity or membrane potential under the permeabilized conditions of the LEAK_PM_ state in all SH-SY5Y cells overexpressing different types of APP relative to the nontransfected control (Figures 2A,B). These results showed that different forms of the APP gene expressed in the cells have different alterations in the level of respiratory capacity and membrane potential, such as abnormally higher expression in cells overexpressing APP without or with a single mutation in the OXPHOS_CI-LINKED_ state but lower expression in cells overexpressing APP with double mutations in the ETS state.

### Treatment With ATF and GTF at Higher Concentrations Increased the Respiratory Capacity and Membrane Potential in SH-SY5Y Cells Overexpressing Different Types of APP

**FIGURE 3:**
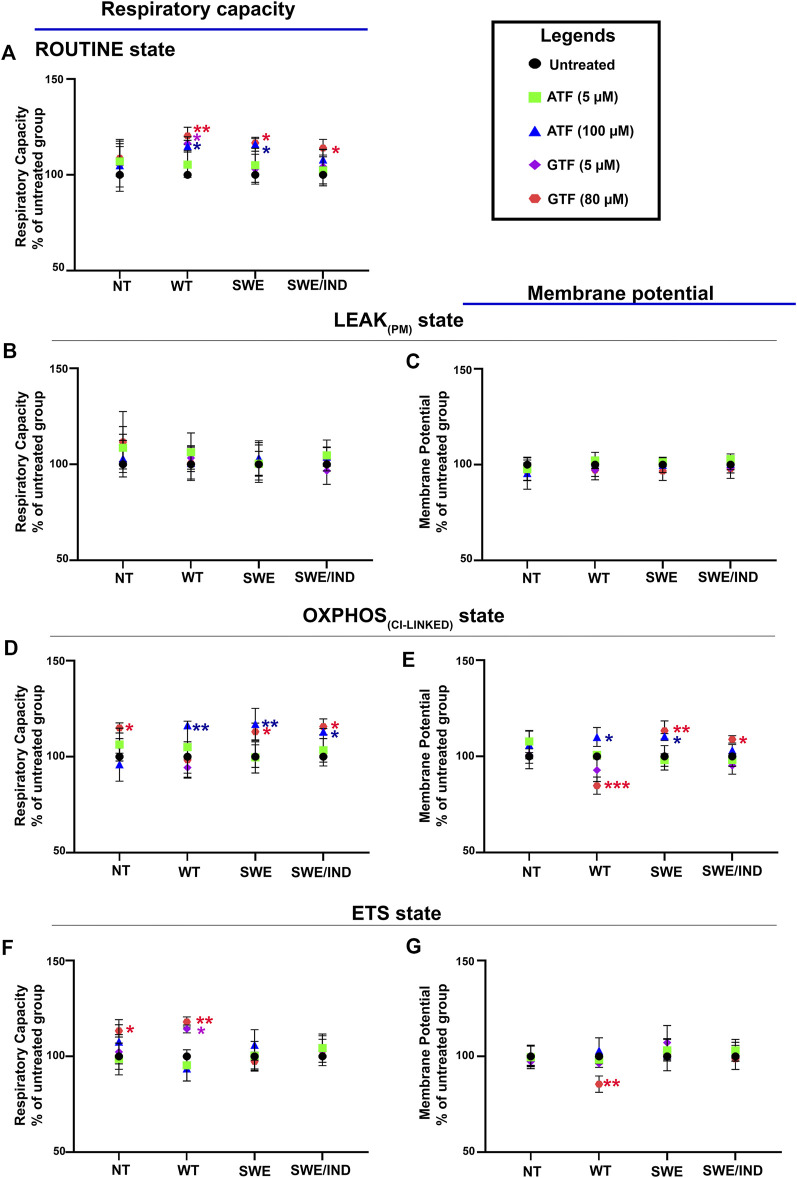
Effects of ATF and GTF treatment on the mitochondrial respiratory capacity and membrane potential in nontransfected SH-SY5Y cells (NT) and cells overexpressing different types of APP (WT, Swe, and Swe/Ind) expressed as % of untreated group in the respective type of cells at **(A)** ROUTINE, **(B, C)** LEAK_PM_, **(D, E)** OXPHOS_CI-LINKED_ and **(F, G)** ETS. *: *p* < 0.05, **: *p* < 0.01, ***: *p* < 0.001. Data are expressed as the mean ± SD, (N = 3).

Our previous data demonstrated that ATF at 100 µM and GTF at 80 µM significantly increased cell viability and decreased the Aβ42 level in SH-SY5Y cells expressing different forms of APP (WT, APP Swe, and APP Swe/Ind) compared to untreated nontransfected controls (Pahrudin Arrozi et al., 2020); therefore, these cells were chosen to further elucidate their effect on the mitochondrial respiratory capacity and membrane potential. The respiratory capacity in the ROUTINE state was significantly increased in APP WT and Swe cells treated with 100 µM ATF compared to their respective untreated controls (Figure 3A). A similar observation was also shown when the same group of cells, including APP Swe/Ind cells, was treated with GTF at 80 µM (Figure 3A). There were no significant differences in the mitochondrial respiratory capacity and membrane potential in the LEAK_PM_ state when all types of cells were treated with ATF or GTF compared to their respective untreated controls (Figures 3B,C).

The mitochondrial respiratory capacity in the OXPHOS_CI-LINKED_ state was significantly increased in all types of APP-overexpressing cells, followed by an increase in the membrane potential in APP WT and Swe cells treated with 100 µM ATF compared to their respective untreated controls (Figures 3D,E). However, treatment with GTF at 80 µM significantly increased the mitochondrial respiratory capacity and membrane potential in the OXPHOS_CI-LINKED_ state in cells overexpressing mutant APP (Figures 3D,E) but significantly decreased the membrane potential in APP WT cells (Figure 3E) compared to their respective untreated controls. Meanwhile, the mitochondrial respiratory capacity and membrane potential in the ETS state were significantly altered with only GTF treatment at 80 µM in APP WT cells compared to the untreated control (Figures 3F,G). These results indicated that treatment with ATF and GTF at higher concentrations modulated the mitochondrial respiratory capacity and membrane potential in different types of APP-overexpressing cells. In addition, lower concentrations of ATF and GTF at 5 µM did not cause any changes to the mitochondrial respiratory capacity and membrane potential, suggesting the dose dependency of the effect of ATF and GTF.

### Treatment With ATF and GTF at Higher Concentrations Increased the Activity of Complex IV Enzyme in SH-SY5Y Cells Overexpressing Different Types of APP

**FIGURE 4:**
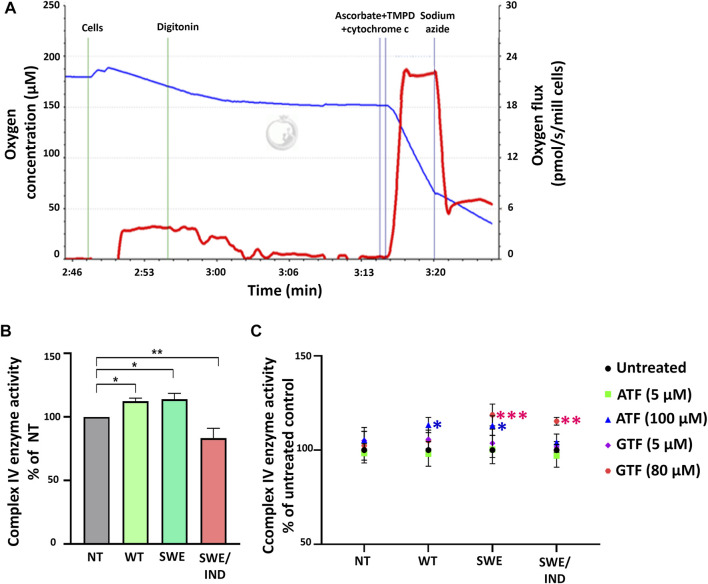
Analysis of complex IV enzyme activity by high-resolution respirometry. **(A)** Qualitative data of complex IV enzyme activity were recorded after the addition of ascorbate, TMPD and cytochrome c expressed as the oxygen flux (pmol/s/mill cells) with chemical background correction using the value recorded after the addition of sodium azide. **(B)** The level of complex IV enzyme activity in SH-SY5Y cells overexpressing different types of APP (WT, Swe, and Swe/Ind) expressed as % of the nontransfected SH-SY5Y control cells (NT). *: *p* < 0.05, **: *p* < 0.01. **(C)** Effects of ATF and GTF treatment on complex IV enzyme activity in nontransfected SH-SY5Y (NT) and cells overexpressing different types of APP (WT, Swe, and Swe/Ind) expressed as % of the untreated group in respective type of cells. *: *p* < 0.05, **: *p* < 0.01, ***p < 0.001. Data are expressed as the mean ± SD, (N = 3).

As oxygen is consumed by respiring cells and its loss was used to measure the rate of respiration, we further investigated the role of ATF and GTF on the key regulatory enzyme responsible for the conversion of oxygen to water molecules. The activity of complex IV was measured using an O2k instrument. Figure 4A demonstrates the graph of complex IV enzyme activity measured as the rate of oxygen consumption (pmol/s/mill cells) shown by the red line. The activity of complex IV was recorded after the addition of ascorbate, TMPD and cytochrome c. Data were corrected using the value recorded after the addition of sodium azide. At the baseline level without treatment with ATF or GTF, the activity of complex IV was higher in SH-SY5Y-APP Wt and SH-SY5Y-APP Swe cells but lower in SH-SY5Y-APP Swe/Ind cells than in nontransfected SH-SY5Y cells (Figure 4B). Treatment with ATF at higher concentrations significantly increased the activity of complex IV enzyme in SH-SY5Y-APP Wt and Swe cells compared to their respective untreated controls (Figure 4C). A similar result was obtained with GTF treatment at higher concentrations, which significantly increased the activity of complex IV enzyme in SH-SY5Y-overexpressing mutant APP forms compared to their respective untreated controls (Figure 4C). These results indicate that ATF and GTF might act as signalling molecules in regulating the complex IV enzyme activation/deactivation pathway.

## Discussion

In this study, we demonstrated that the mitochondrial respiratory capacity and membrane potential were abnormally higher in APP WT or Swe cells, particularly in the OXPHOS_CI-LINKED_ state, but lower in APP Swe/Ind cells, particularly in the ETS state. A similar observation was also shown for complex IV enzyme activity, which is the major regulator of oxidative phosphorylation; it was higher in APP WT or Swe cells but lower in APP Swe/Ind cells. We also demonstrated that treatment with ATF and GTF at higher concentrations modulated the respiratory capacity and membrane potential, particularly in the ROUTINE and OXPHOS_CI-LINKED_ states, as well as complex IV enzyme activity in APP-overexpressing cells.

We assessed mitochondrial oxidative metabolism using a cell suspension instead of isolated mitochondria, as this required a mild detergent, such as digitonin, to selectively permeabilize the cell plasma membrane *via* interaction with cholesterol, thus allowing free exchange of organic molecules and inorganic ions between the cytosol and the immediate cell environment while maintaining the integrity and localization of organelles, the cytoskeleton, and the nucleus. In the preparation of isolated mitochondria, however, the mitochondria are separated from other cell fractions and purified by differential centrifugation, entailing the loss of mitochondria; thus, this might compromise the mitochondrial yield or structural and functional integrity (Gnaiger, 2020).

The different alterations in the mitochondrial respiratory capacity and membrane potential were probably influenced by the types of APP accumulated in the cells. It has become generally accepted that the accumulation of Aβ is responsible for mitochondrial impairment (Cieślik et al., 2020; Quintana et al., 2020; Zhang et al., 2018); however, there is also evidence for the relationship between APP processing and mitochondrial bioenergetic regulation (Puig and Combs, 2013; Wilkins and Swerdlow, 2017). Our data highlight the need for a better understanding of the cellular function of APP and its metabolites, and further work is needed to elucidate their potential contribution to the alteration of mitochondrial bioenergetics. A study by Reddy et al. (Reddy et al., 2004) demonstrated the upregulation of gene expression involved in the regulation of mitochondrial metabolism in a Tg2576 transgenic mouse model transfected with the APP Wt gene as early cellular changes during the progression of Alzheimer’s disease (Swerdlow, 2018). Meanwhile, a study by Hauptmann et al. (Hauptmann et al., 2009) showed a decreased mitochondrial respiration rate in the ETS state followed by a decrease in the mitochondrial membrane potential, complex IV enzyme activity and ATP levels in isolated mitochondria of transgenic mouse models carrying Swedish and London APP mutations (similar mutation position to Indiana mutation with different amino acid substitutions) as late cellular changes in disease progression.

**FIGURE 5:**
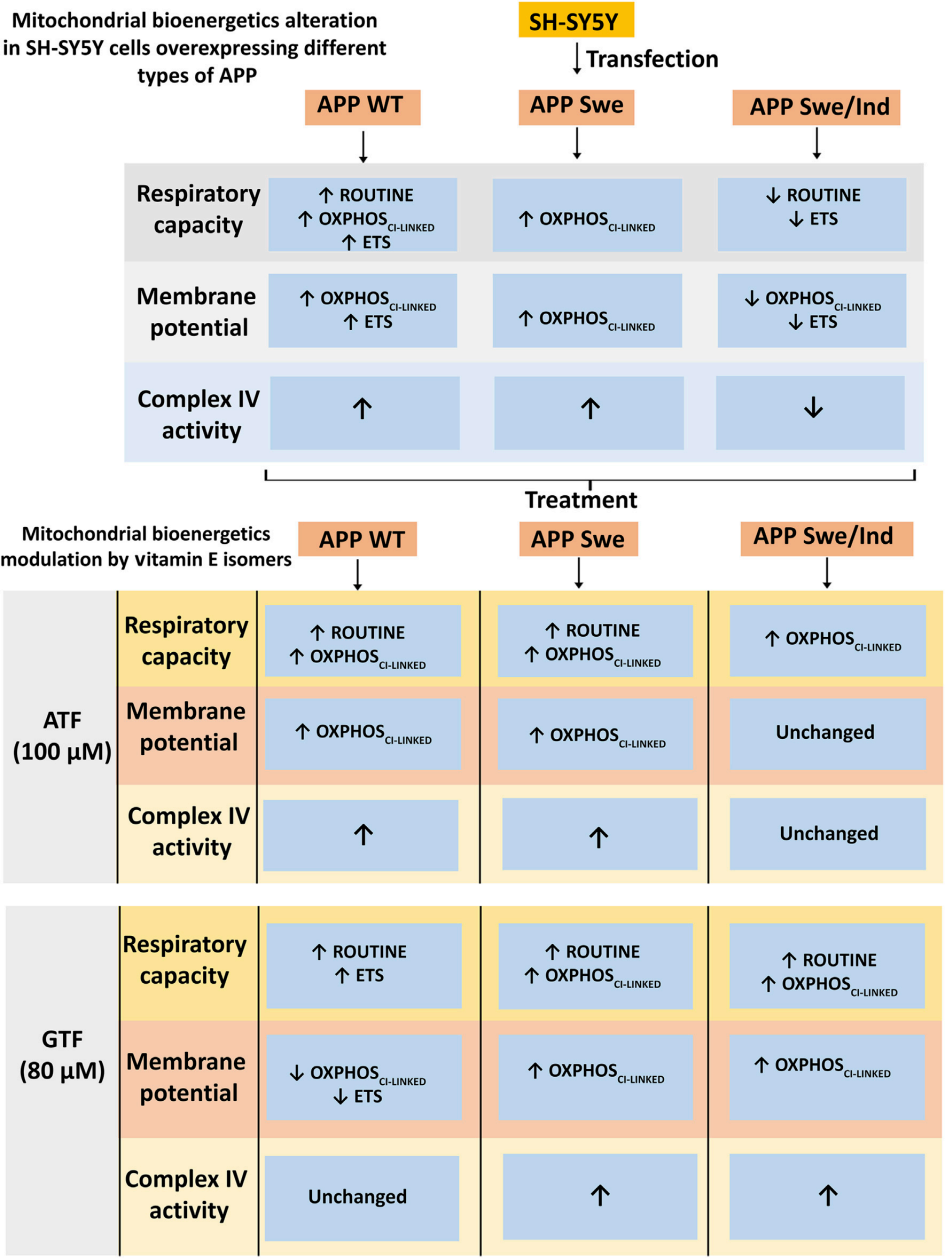
Schematic diagram of mitochondrial bioenergetics alteration in SH-SY5Y cells overexpressing different types of APP and modulation by vitamin E isomers. The APP WT and Swe overexpressing cells showed increased in mitochondrial bioenergetics at OXPHOS_CI-LINKED_ state accompanied by an increased in complex IV enzyme activity whereas the APP Swe/Ind overexpressing cells showed decreased in mitochondrial bioenergetics at ETS state followed by a decreased in complex IV enzyme activity. ATF and GTF modulated mitochondrial bioenergetics differently in different types of APP overexpressing cells generally by increasing the respiratory capacity, membrane potential and complex IV enzyme activity.

The subsequent mechanism of action of ATF or GTF in this study was unclear, as the gene or protein expression involved in mitochondrial bioenergetic regulation was not measured. We speculated that ATF or GTF might act as signalling molecules in the transcriptional alteration of mitochondrial genes involved in mitochondrial energy metabolism, as shown by the increased/decreased complex IV enzyme activity in different types of APP-overexpressing cells (Figure 5). A previous study demonstrated that aged mice supplemented with different antioxidants, including ATF, modulate age-related changes in the activity of complex enzymes in the cerebral ETS composed of complexes I, II, III and IV (Sharman and Bondy, 2001). Furthermore, oral treatment with a combination of ATF and folic acid in an *in vivo* model of AD also modulated mitochondrial complex activity, especially complexes I and IV (Figueiredo et al., 2011). In addition, the gene expression profiles obtained in the brains of aged mice supplemented with ATF or a mixture of ATF and GTF showed a significant modulation effect by the combination of both isomers but not ATF alone on genes involved in mitochondrial energy metabolism, including ATP synthesis, which is primarily dependent on oxidative phosphorylation (Park et al., 2008), thus suggesting that the combination of vitamin E isomers might have better modulation than a single isomer. Moreover, ATF supplementation in aged rats was restored in a dose-dependent manner, and the decreases in tissue and mitochondrial respiration as well as complex I and IV activities in both the hippocampus and frontal cortex region (Navarro et al., 2011) supported the modulatory effect of ATF on mitochondrial bioenergetics.

There are several limitations in the present study. First, an *in vivo* study was not included to further confirm the role of ATF and GTF in modulating mitochondrial oxidative metabolism in a higher model system. Second, the expression of the genes or proteins involved in the regulation of mitochondrial oxidative metabolism was also not measured to further elucidate the mechanism of action of ATF or GTF. Third, only complex IV enzyme was measured, as this is the major enzyme regulator in ETS that permits the transfer of electrons from reduced cytochrome c to the final acceptor of electrons, oxygen, which is important to characterize the function of mitochondrial respiration.

## Conclusion

Both ATF and GTF modulated the mitochondrial respiratory capacity and membrane potential as well as the activity of the complex IV enzyme in different types of APP-overexpressing cells. Although preliminary, these findings may indicate the possible health benefits of ATF or GTF and, in part, may contribute to planning for a strategy of utilizing vitamin E isomers against mitochondrial-related diseases such as AD.

## Data Availability

The original contributions presented in the study are included in the article/Supplementary Material, further inquiries can be directed to the corresponding author.
