# Supplementary material for: Modulatory Effects of Alpha- and Gamma-Tocopherol on the Mitochondrial Respiratory Capacity and Membrane Potential in an In Vitro Model of Alzheimer’s Disease
Source: Front Pharmacol. 2021 Nov 22;12:698833. doi: 10.3389/fphar.2021.698833 (PMC8646103; doi:10.3389/fphar.2021.698833)
Supplement: Supplementary file 1 [file DataSheet1.pdf]

Supplementary Material

1 Supplementary Figures and Tables

1.1 Supplementary Figures

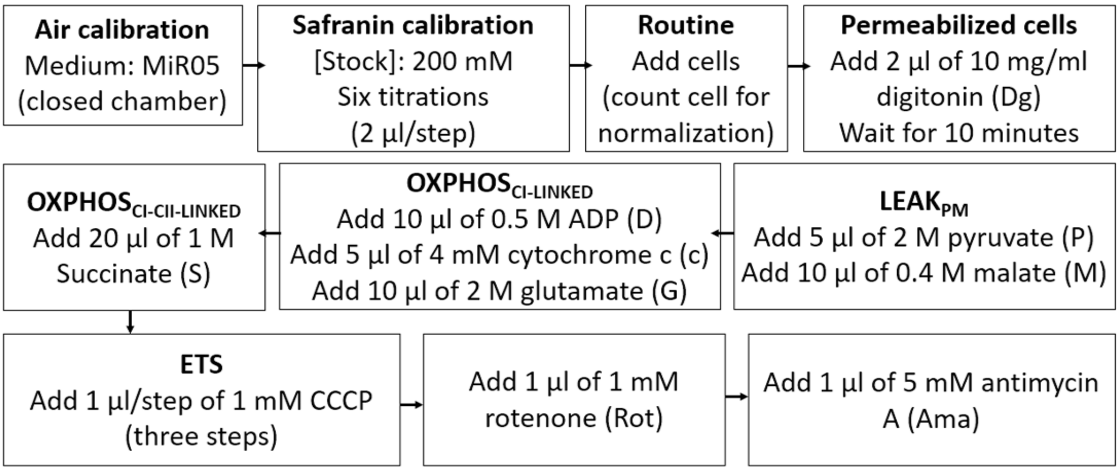

Supplementary Figure 1. The substrate–uncoupler–inhibitor titration (SUIT) protocol for simultaneous determination of mitochondrial respiration rate and membrane potential.

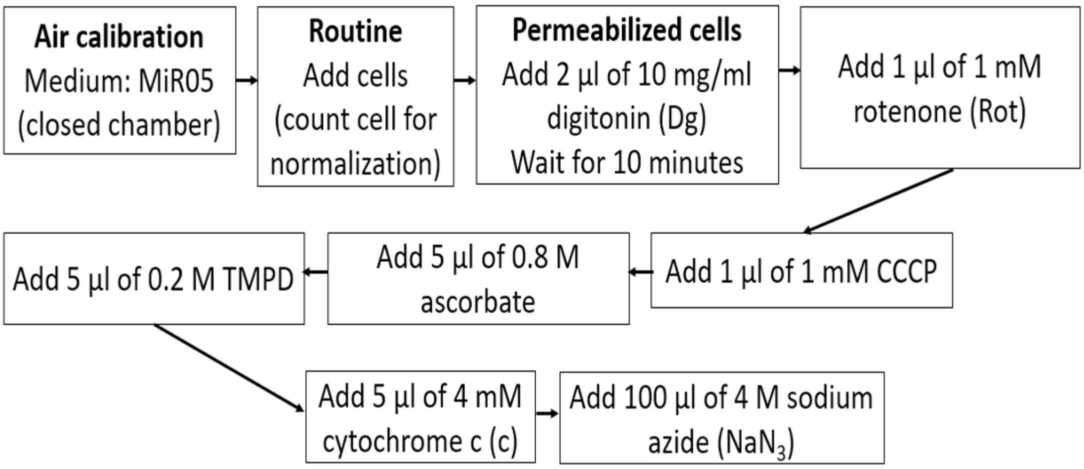

Supplementary Figure 2. The Protocol for determination of complex IV enzyme activity by high resolution respirometry.

1.2 Supplementary Tables

**Substances**	**weight (mg)**	**Volume of solvent**	**Final stock Concentration**	**Storage (˚C)**
Digitonin	10	1 ml DMSO	10 mg/ml	-20
Safranin	3.5	50 ml H_2O	200 µM	25
#Pyruvate	44	0.2 ml H_2O	2 M	-
*Malate	536	10 ml H_2O	0.4 M	-20
*Glutamate	3382	10 ml H_2O	2 M	-20
*ADP	501	2 ml H_2O	0.5 M	-80
CCCP	1.02	5 ml DMSO	1 mM	-20
Rotenone	3.94	10 ml EtOH	1 mM	-20
Antimycin A	11	4 ml EtOH	5 mM	-20

*pH was adjusted to 7.1
Freshly prepared

Supplementary Table 1. Preparation of chemical substances for SUIT protocol

Chemicals	Formula weight (g/mol)	Weight or volume	Final concentration
EGTA	380.4	0.190 g	0.5 mM
$MgCl_2.6H_2O$	203.3	0.610 g	3 mM
Lactobionic acid	358.3	120 ml from 0.5 M stock solution	60 mM
Taurine	125.1	2.502 g	20 mM
KH_2PO_4	136.1	1.361 g	10 mM
HEPES	238.3	4.77g	20 mM
D-sucrose	342.3	37.65 g	110 mM
BSA	-	1 g	1 g/l

Supplementary Table 2. Preparation 1 L of Mir05 solution. To prepare 1 L Mir05 solution, all the above materials (except lactobionic acid and BSA) were weighted and put in the 1 L of Schott bottle. A total of 800 ml of Milli Q water was added and stirred well using magnetic stirrer at 30°C. Then, a total of 120 ml of lactobionic acid was added and the pH was adjusted to 7.1. The BSA was dissolved separately in a beaker to prevent bubble formation and added slowly into the mixture while stirred soft. The Mir05 solution was aliquoted into 15 mL Falcon tubes and kept at -20 °C until use.

Substances	weight (mg)	Volume of solvent	Final stock concentration	storage (˚C)
Digitonin	10	1 ml DMSO	10 mg/ml	-20
Rotenone	3.94	10 ml EtOH	1 mM	-20
CCCP	1.02	5 ml DMSO	1 mM	-20
*#Ascorbate	1584	10 ml H_2O	800 mM	-20
#TMPD	47.4	1 ml H_2O	200 mM	-20
Cytochrome c	50	1 ml H_2O	4 mM	-20
NaN_3	260	1 ml H_2O	4 M	-20

*pH was adjusted to 7

Protected from light

Supplementary Table 3. Preparation of substances for complex IV enzyme activity
